# Supplementary figures and images for: Anti-CD19 CAR T cells potently redirected to kill solid tumor cells
Source: PLoS One. 2021 Mar 18;16(3):e0247701. doi: 10.1371/journal.pone.0247701 (PMC7971483; doi:10.1371/journal.pone.0247701)

S1 Fig.

A)

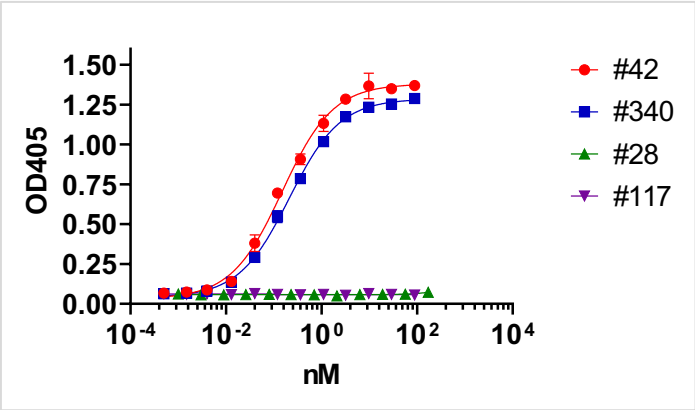

B)

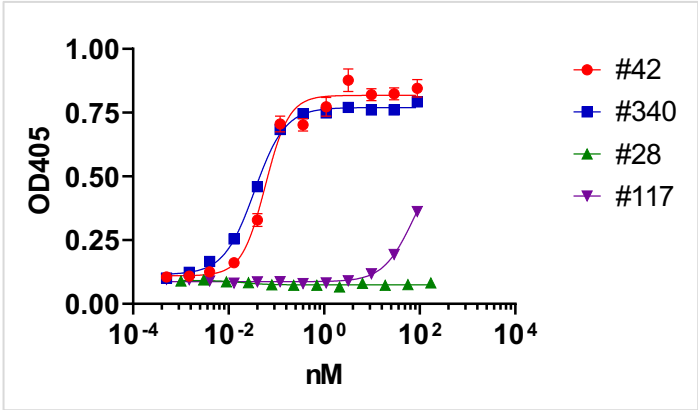

Supplement: S1 Fig — A) The capture agent was Her2-Fc and the detection agent was anti-CD19 antibody FMC63. B) ELISA assay of bridging protein binding: the capture agent was anti-CD19 antibody FMC63 and the detection agent was biotinylated Her2. In both assays the bridging proteins (#42, #340) bound significantly better than the control proteins (#28, #117); p < 0.01 at the bridging protein EC50 value. (PDF) [file pone.0247701.s001.pdf]

S2 Fig.

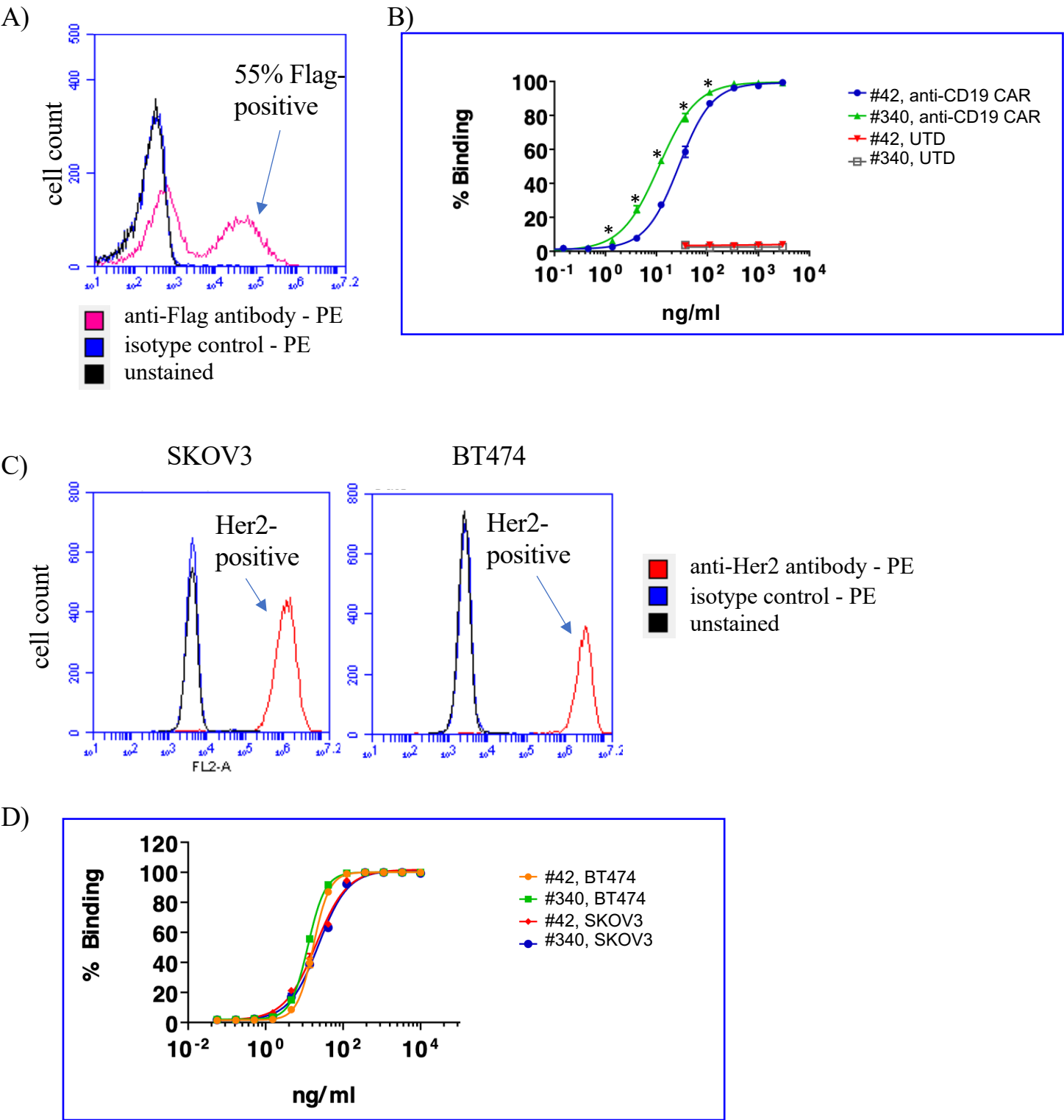

Supplement: S2 Fig — A) Anti-Flag-tag staining of CAR-CD19 transduced donor primary T cells (donor 69, results are representative of n = 4 transduction experiments). B) Binding of purified CD19-anti-Her2 bridging proteins to anti-CD19 CAR transduced primary T cells (donor 69, n = 2, * p < 0.004 for binding of #42 vs #340). C) Expression of antigen Her2 on the ovarian carcinoma cell line SKOV3 (results are representative of n = 6 flow cytometry assays). D) Binding of bridging proteins to SKOV3 cells (triplicate wells, the data are from 1 of 2 experiments performed). (PDF) [file pone.0247701.s002.pdf]

S3 Fig.

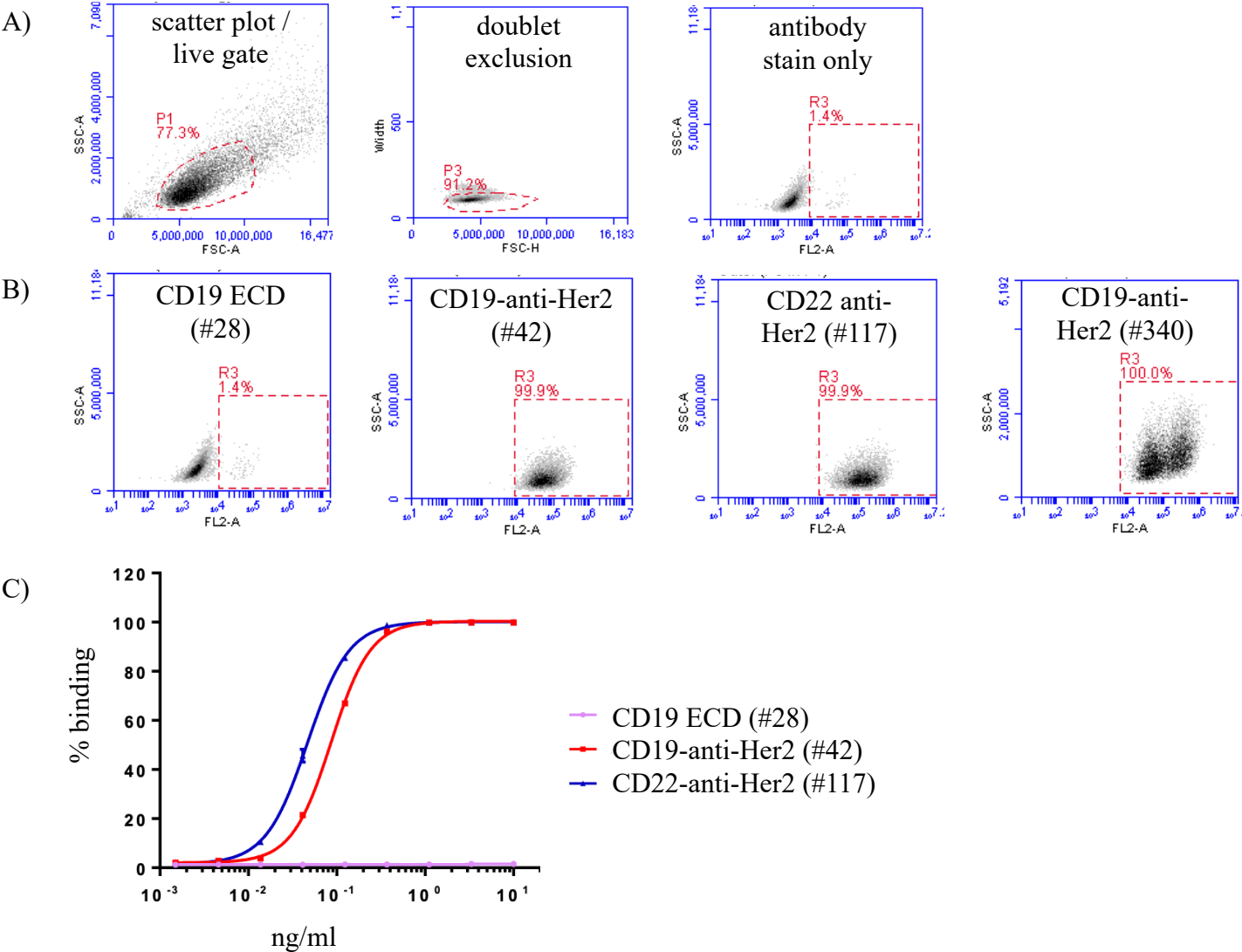

D)

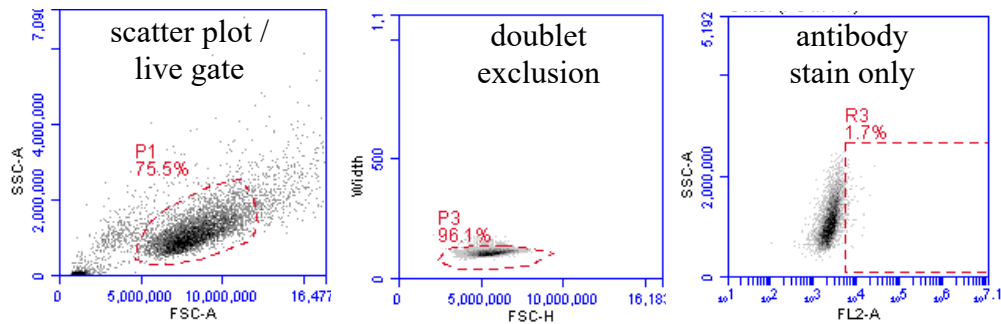

E)

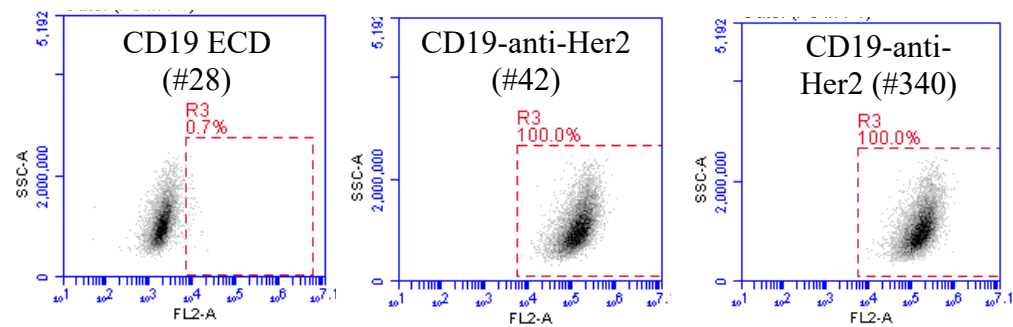

F)

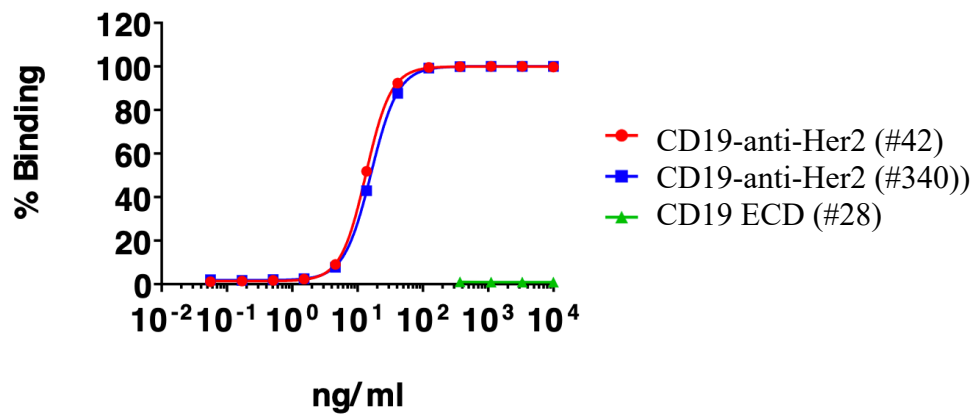

Supplement: S3 Fig — A) Representative gating of SKOV3 cells. B) Binding with 1 μg/ml of control and bridging proteins to SKOV3 cells. C) dose response binding to SKOV3 cells. D) Representative gating of BT474 cells. E) Binding with 1 μg/ml of control and bridging proteins to SKOV3 cells. F) dose response binding to BT474 cells. Detection of bound protein was with anti-CD19-PE antibody except for the CD22-anti-Her2 samples that were detected with anti-CD22-PE antibody. (PDF) [file pone.0247701.s003.pdf]

**S4 Fig.**

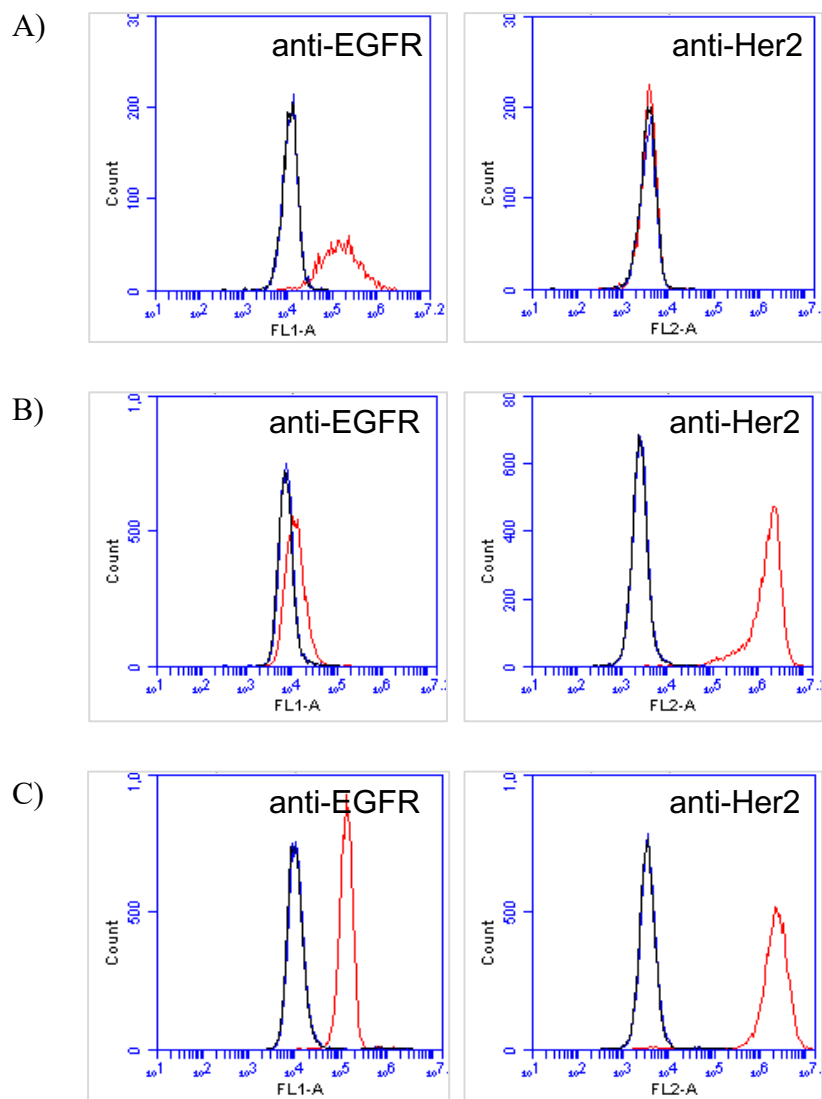

Supplement: S4 Fig — Analyses of Her2 and EGFR expression on cell lines. A) K562-EGFR cells. B) BT474 cells. C) SKOV3 cells. (PDF) [file pone.0247701.s004.pdf]

S5 Fig.

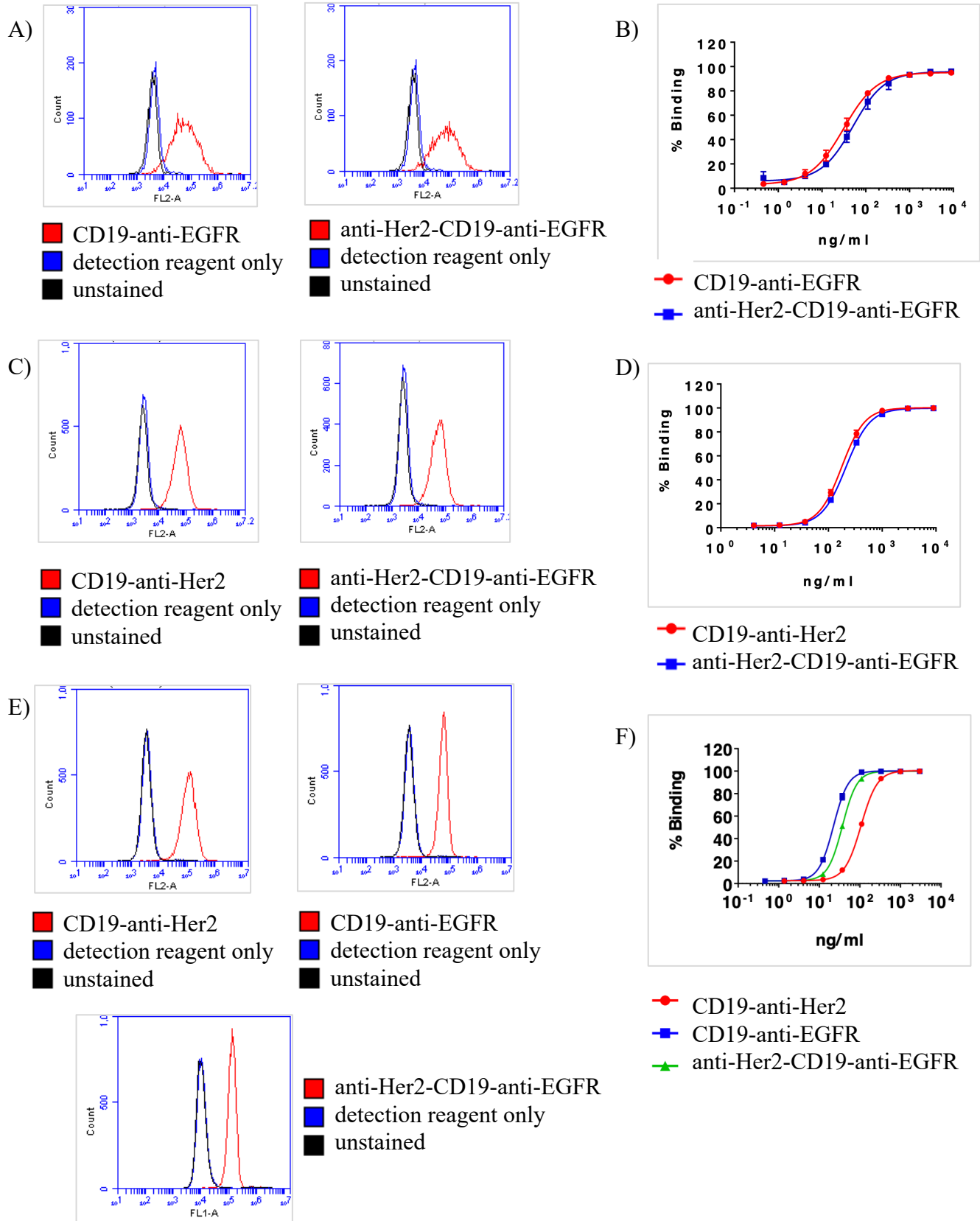

Supplement: S5 Fig — A) Flow cytometry data showing various bridging proteins binding at saturation (A, C, E) and in dose response curves (B, D, F) to K562-EGFR cells (A, B), BT474 cells (C, D) and SKOV3 cells (E, F), as detected with anti-CD19 antibody FMC63-PE. (PDF) [file pone.0247701.s005.pdf]

S6 Fig.

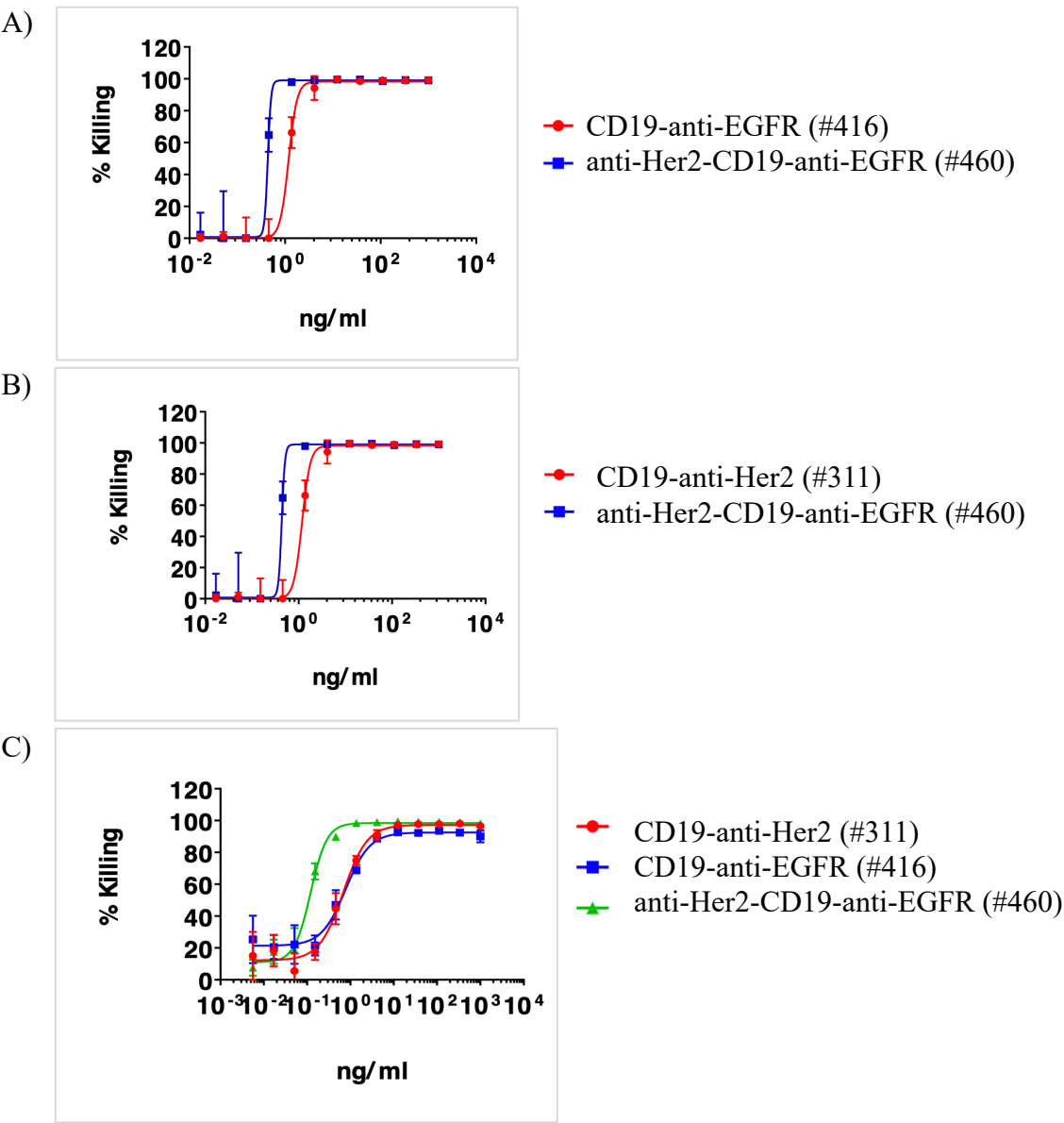

Supplement: S6 Fig — A) K562-EGFR. B) BT474. C) SKOV3. Anti-CD19 CAR T cells (donor 54, 47% Flag-tag positive) were added at an E:T ratio of 10:1. Bridging proteins were added in a dose titration into the cytotoxicity assay. (PDF) [file pone.0247701.s006.pdf]

S7 Fig.

A)

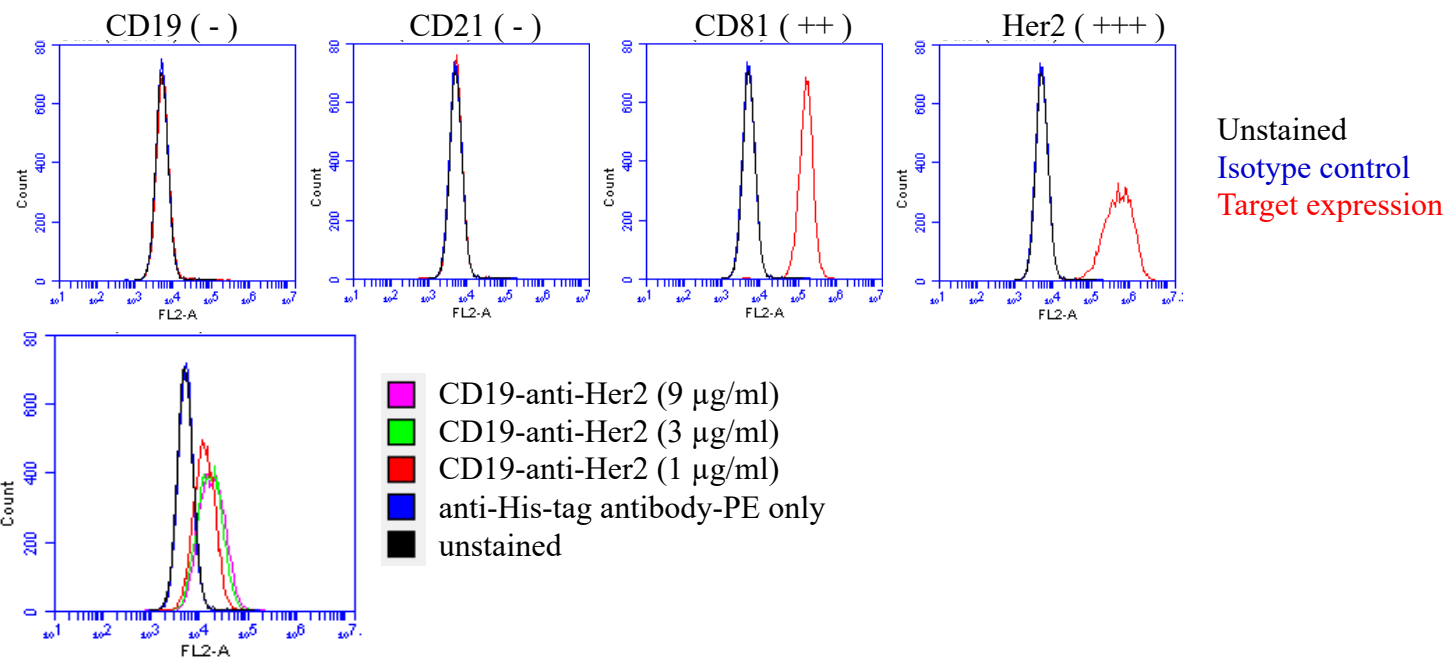

B)

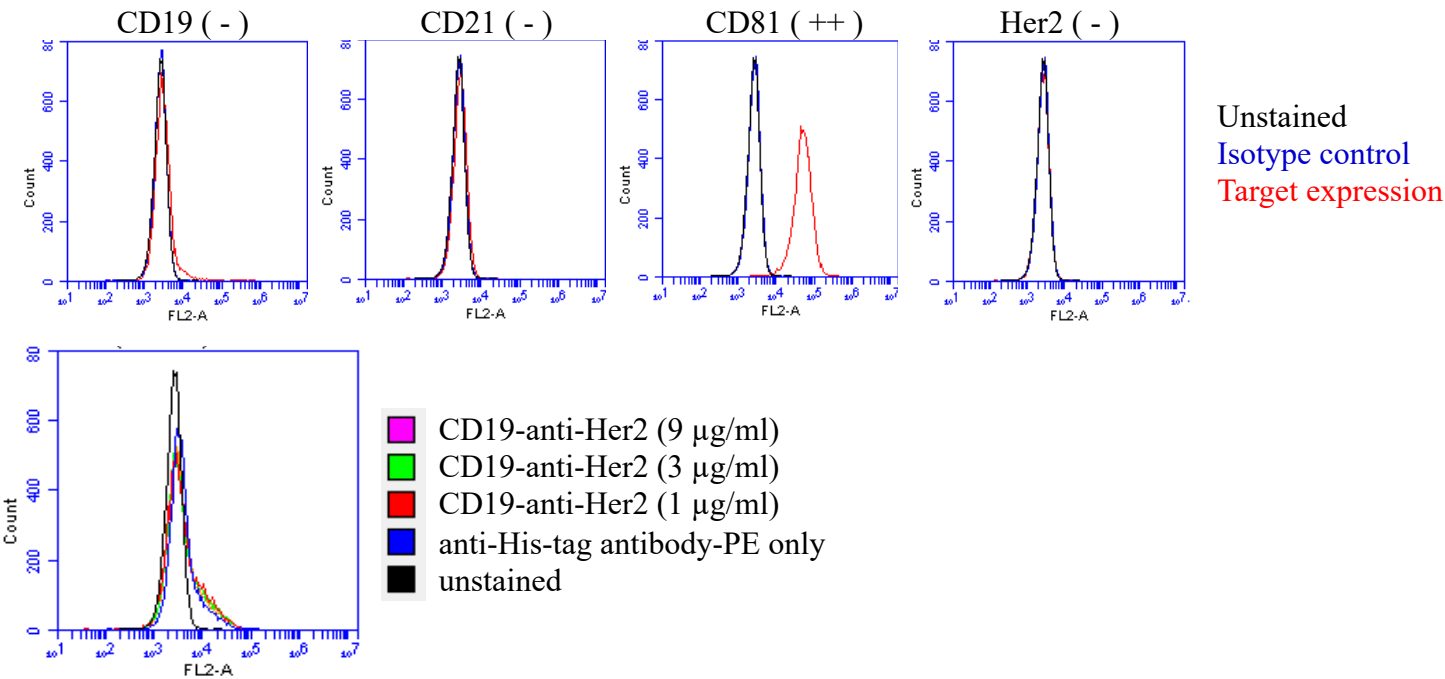

C)

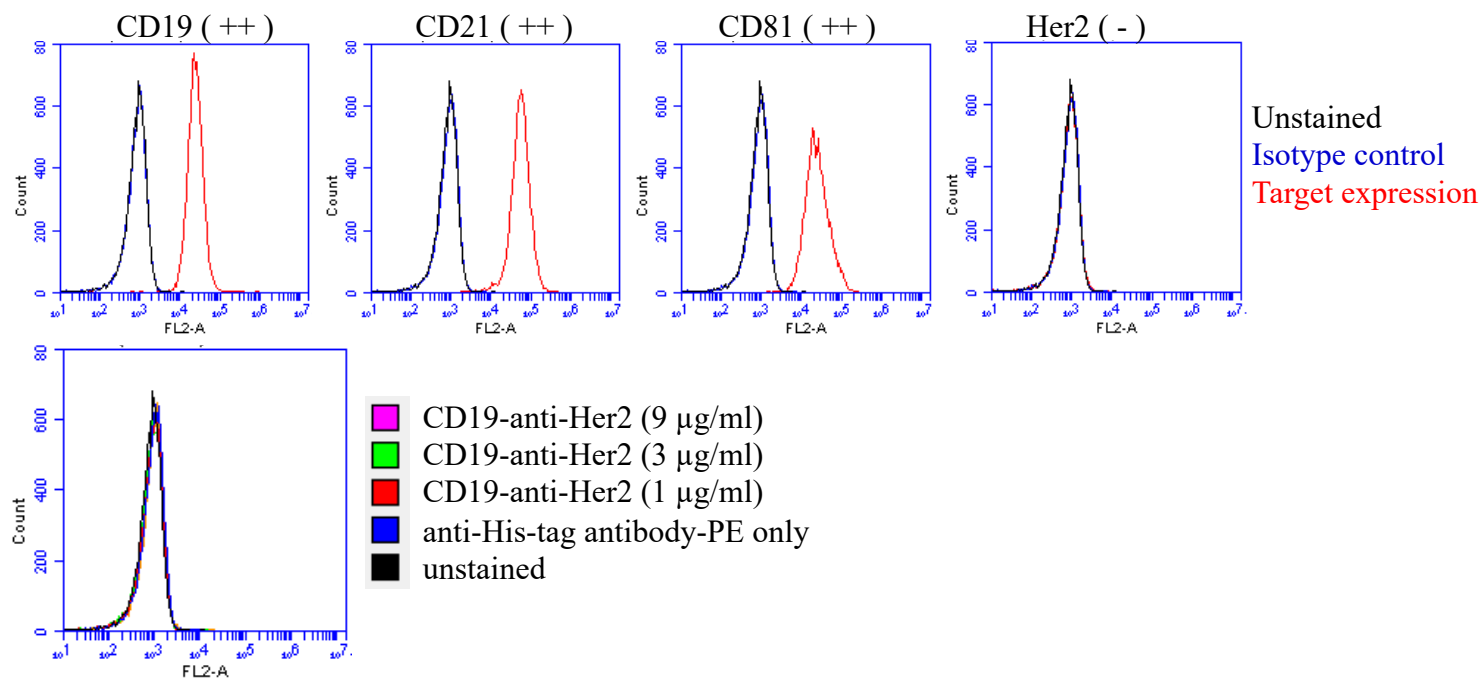

D)

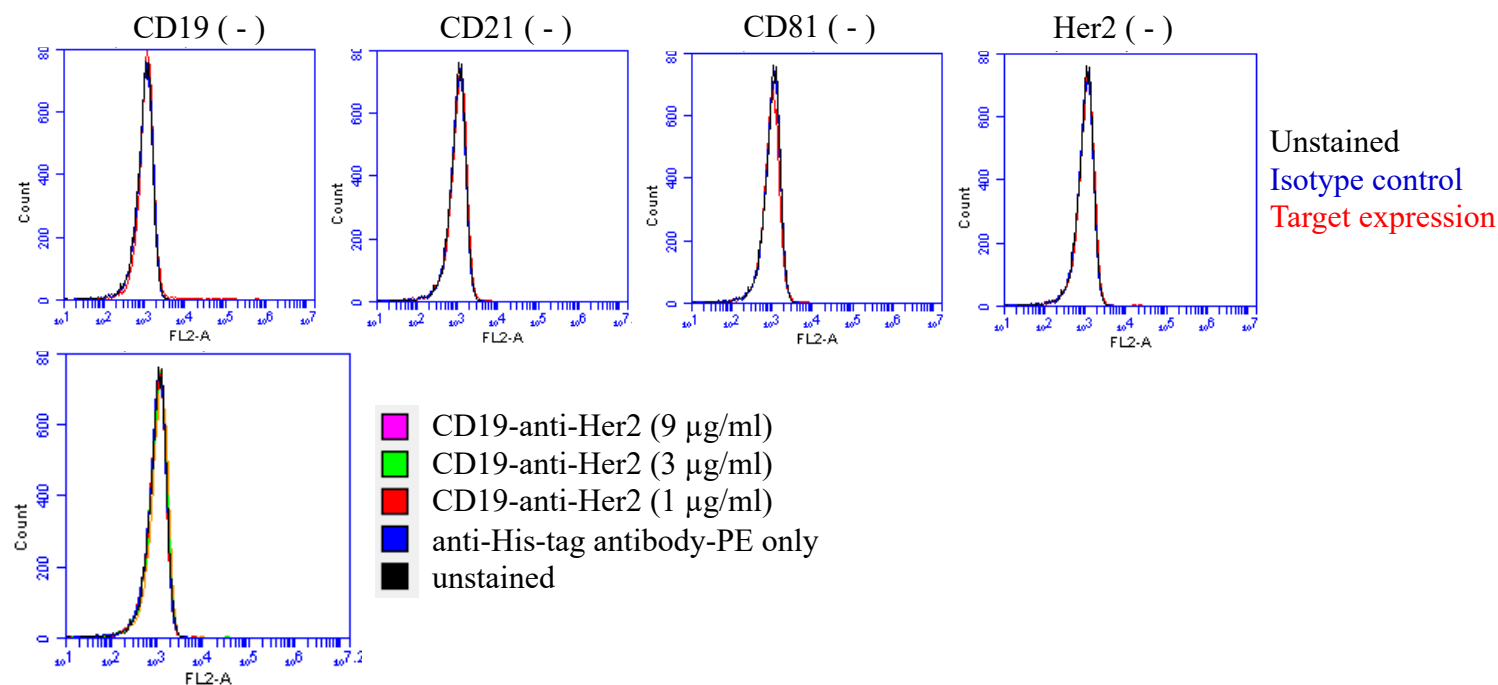

E)

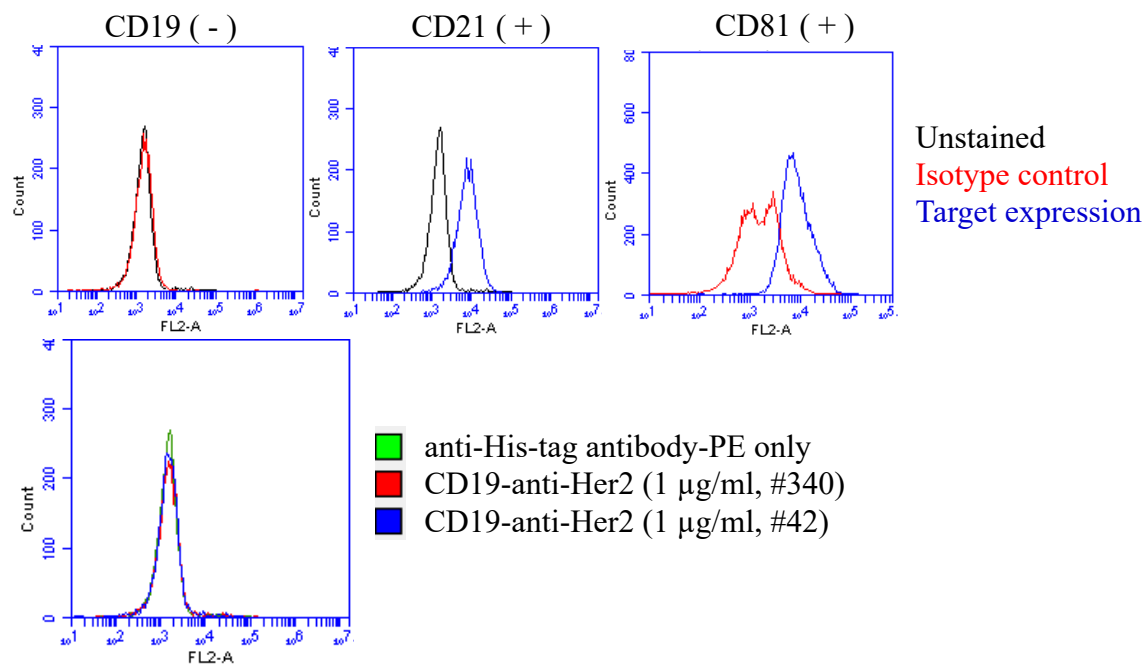

Supplement: S7 Fig — A) SKOV3. B) K562. C) Raji. D) U937. E) OCI-LY3. (PDF) [file pone.0247701.s007.pdf]

S8 Fig.

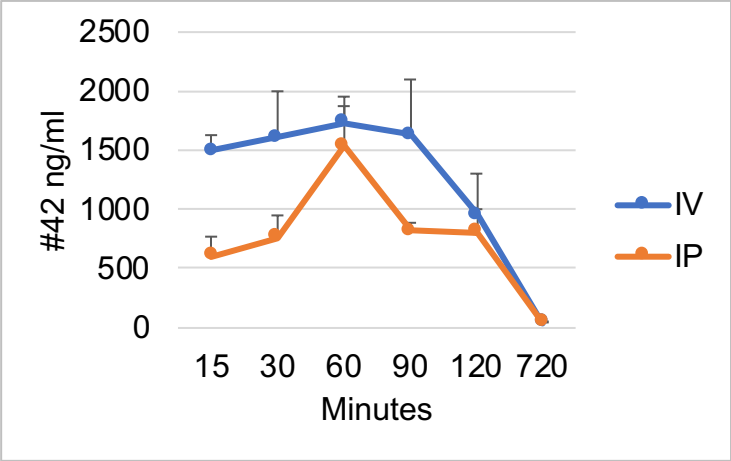

Supplement: S8 Fig — PK measurements of a CD19-anti-Her2 bridging protein after injection into Rag-/- common gamma-/- mice. (PDF) [file pone.0247701.s008.pdf]

S9 Fig.

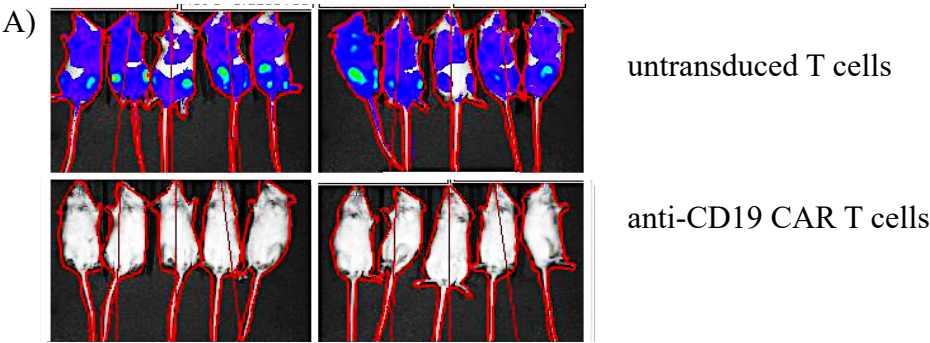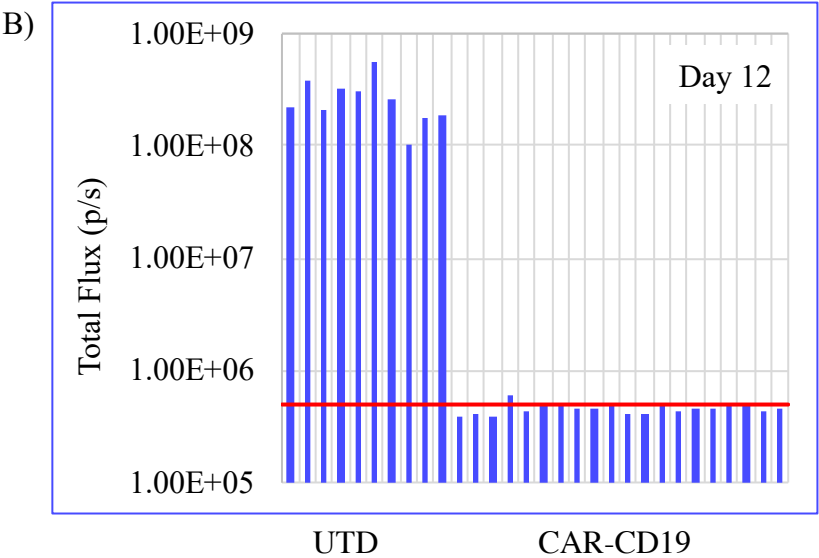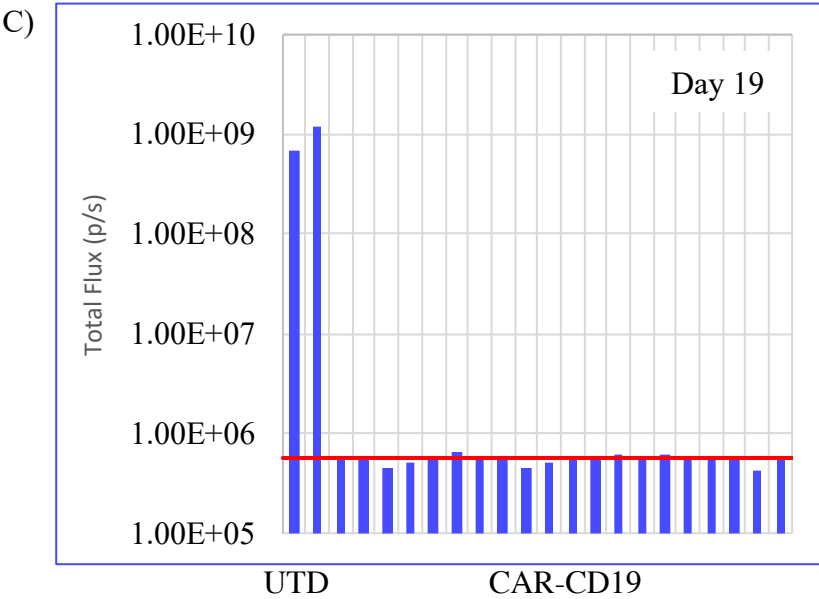

Supplement: S9 Fig — Mice were implanted with Nalm6 cell IV, then treated 4 days later with anti-CD19 CAR T cells (donor 54, 99.5% CD3-positive, 52% Flag-tag positive for CAR expression, and 56% CD8 positive by flow cytometric analyses). A) Representative images, day 12. B) Luminescence measurement, day 12. C) Luminescence measurement, day 19: note only 2 control (UTD) mice were still alive at day 19. The red bar marks the background luminescent reading. UTD lumin readings were statistically higher than treated mice on both days 12 and 19 p < 0.001. This experiment is representative of 3 independent experiments. (PDF) [file pone.0247701.s009.pdf]
