# Supplementary material for: Anti-CD19 CAR T cells potently redirected to kill solid tumor cells
Source: PLoS One. 2021 Mar 18;16(3):e0247701. doi: 10.1371/journal.pone.0247701 (PMC7971483; doi:10.1371/journal.pone.0247701)
Supplement: S1 Table — The long dashes indicate a linker sequence. P2A refers to a cleavage sequence. BP refers to a bridging protein. (PDF) [file pone.0247701.s010.pdf]

S1 Table. List of additional constructs used, with a description of the encoded sequences in column 2 and the format in which the expressed sequence is utilized in column 3. The long dashes indicate a linker sequence. P2A refers to a cleavage sequence. BP refers to a bridging protein.

Construct #	Description	Modality
**28**	CD19 ECD	Purified protein
**42**	CD19 ECD – anti-Her2	Purified BP
**117**	CD22 ECD – anti-Her2	Purified BP
**142**	Sequence of 254 – P2A – sequence of 42	CAR-CD19 + BP
**311**	Stabilized CD19 ECD – anti-Her2	Purified BP
**416**	Stabilized CD19 ECD – anti-EGFR	Purified BP
