# Supplementary material for: Anti-CD19 CAR T cells potently redirected to kill solid tumor cells
Source: PLoS One. 2021 Mar 18;16(3):e0247701. doi: 10.1371/journal.pone.0247701 (PMC7971483; doi:10.1371/journal.pone.0247701)
Supplement: S2 Table — Antigen expression is indicated as negative (-), dim (+), bright (++), or very bright (+++) or not done (nd). Binding of bridging proteins to cell lines was analyzed by flow cytometry as described in the text. The results are summarized as positive (+) or negative (-) for detectable binding. (PDF) [file pone.0247701.s011.pdf]

S2 Table. Summary of bridging protein binding to cell lines. Antigen expression is indicated as negative (-), dim (+), bright (++), or very bright (+++) or not done (nd). Binding of bridging proteins to cell lines was analyzed by flow cytometry as described in the text. The results are summarized as positive (+) or negative (-) for detectable binding.

cell line	antigen expression					bridging protein binding to cell lines	
	CD19	CD20	CD21	CD81	Her2	#42	#340
SKOV 3	-	-		++	+++	+	+
K562	-	-		++	-	-	-
Raji	++	++	++	++	-	-	-
U937	-	-	-	-	-	-	-
OCI-Ly3	-	++	+	+	nd	-	-
