# Supplementary material for: Anti-CD19 CAR T cells potently redirected to kill solid tumor cells
Source: PLoS One. 2021 Mar 18;16(3):e0247701. doi: 10.1371/journal.pone.0247701 (PMC7971483; doi:10.1371/journal.pone.0247701)
Supplement: Attachment — Submitted filename: Response to Reviewers.docx [file pone.0247701.s013.docx]

**Response to Reviewers**

We thank the editor and reviewers for their extensive and helpful comments, we believe the paper to be much improved.

**Reviewer #1:**

Malcom Brenner´s group published in 2007, where latent EBV infection was suggested as persisting source for stimulating EBV-specific T cells modified with anti-CD30 CAR.

**We thank the reviewer for pointing these out. Please see references 34 and 35, added.**

Why employing a system to eradicate tumors, which knock out the B cell compartment and therefore potential virus-neutralizing antibody responses? The authors should include limitations of their approach (including obvious regulatory hurdles).

**B cell aplasia is a well understood and generally readily managed consequence of anti-CD19 CAR T cell therapy, as it is of rituximab therapy etc. There have been no regulatory concerns raised by the agency. We have however added a discussion of B cell aplasia to page 25.**

please more stringent presentation (for example present data containing only the improved CD19-anti-HER2 binding protein (#340) instead of #42 and #340, skip data showing less suitability of BP aggregates.

**We agree and have greatly streamlined the manuscript and presentation and have removed non-essential sections such as the aggregation assay data.**

In almost all datasets, the number of replicates or independent experiments is not clear to the reader.

**We have added the number of biologic replicates (eg. # of wells) and also the number of times an experiment was repeated, throughout the paper.**

Major points

The authors performed transductions of T cells resulting in different transduction efficiencies. They did not sort CAR-positive T cells nor did they perform antibiotic selection of transduced T cells. However, they used specific effector to target ratios in the cytotoxicity assays depicted in figures 1 and 2. Please clarify, if the effector to target ratio is calculated by the number of T cells or by the number of CAR-positive T cells, the latter resulting in a significant number of non-transduced T cells in the experiments. How were the non-transduced T cell (UTD control) treated, how was their number adjusted in the experiments? The authors should clarify the points.

**We used total T cell count throughout, not adjusted for CAR-% percentage. We have added details about the CAR preparations used in the different experiments.**

What is the purity of the expanded CAR-T cell populations? Have the authors ruled out NK cell or B cell contaminations in all experiments? It is advised to provide data showing purity of the expanded/restimulated immune cells.

**All CAR T cell preps are 99% or greater purity: NK cells and B cells are removed during the initial T cell separation.**

Furthermore, since re-stimulation of CD19-CAR T cells equipped with ectopic binding protein expression or recombinant binding protein is the centerpiece of the manuscript and might have clinical impact, it is recommended to recapitulate expansion of T cells transduced with #374, #416, #460 using autologous B cells as source for CD19.

**CAR T cells are prepared from incoming donors and stored until use. Donor B cells will not freeze well and die overnight in culture. We would like to do more experiments like you describe but it is logistically very difficult.**

It is furthermore recommended to demonstrate the selective expansion of CAR-positive T cells (increase in percentages in the T cell population) upon stimulation with CD19+ target cells and the absolute increase in CAR-T cell numbers (please provide absolute numbers and no normalized values as depicted in Fig. 3.).

**We have provided absolute numbers for the fold increase, and thank you for this excellent suggestion.**

Another concern relates to the cytotoxicity assay chosen for the experiments. The authors employs Luc-modified target cells for analysis. Target cells were confronted with effector cells and binding proteins for 48h. Such a setting should result in exaggerated Luc activity in control cells due to cell doublings when compared to treated target cells, which might have succumbed to cells death during the first hours of the assay. Likewise, this results in an overstated cytotoxicity of effector cells towards target cells. It is recommended to shorten the incubation time of the cytotoxicity assay to 18- 24 h.

**We do not obtain robust results in such a short time – such assays are best suited to kinetic analyses, which equipment we cannot afford.**

Calculate specific lysis of cells by including spontaneous(minimal) lysis and maximal lysis of untreated target cells and to calculate specific cytotoxicity to the formula specific cytotoxicity = 100 × (Luc activity target cells − Luc activity minimal release) / (Luc activity maximum release − Luc activity minimal release).

**All the controls are shown.**

In this regard, please provide data, showing that treatment with CD19 bridging proteins alone to HER2 and EGFR or supernatants derived from T cells transduced with # 142 and #374 do not affect proliferation or viability of antigen-positive target cells. Therefore, the authors are strongly encouraged to exclude a potential therapeutic effect of the bridging proteins, which also might have implications for in vivo experiments.

**This is an important point to understand – the bridging proteins are small (50kD) proteins with no inherent activity. The CD19 does not bind to anything. The scFv binds to Her2 but is inactive. There is no Fc domain so they cannot attract immune cells. These are monomers so they cannot signal. We have done the order of additional assays to show no effect of bridging protein on the relevant cells.**

The authors suggest that the CD19-anti-HER2 bridging protein does not compromise CD19-CAR- function. Yet, when adding a competing ligand for a CAR most readers likely anticipate that the cytotoxicity of CD19-CAR T cells towards CD19+ NALM6 target cells should be attenuated. The authors should give reasons why a competing CD19 bridging protein does not inhibit CD19-CAR T cell cytotoxicity towards NALM6 cells (i.e. crosslinking on target cells increasing avidity etc.).

**Thank you for asking this important question. This is a simple matter of mass action. Only a small fraction of the CAR domains expressed on a CAR T cell need to be engaged in order to trigger cytotoxicity. This is why the IC50 is always a log or more to the left of the EC50. Also we have clearly demonstrated no competition by the bridging protein in the order of addition assay.**

Minor points:
Fig.1D and E, what is the effector to target ratio used for the experiments?

**Thank you, we have added the CAR T cell information.**

Supplemental Figure S3 A, for clarification, please provide dot blots showing double staining of CAR and bound #42 and #340 recombinant proteins, respectively.

**Thank you, we have added additional FACs data throughout.**

**Reviewer #2:**

Major points:

1) The authors should discuss major drawbacks of their approach. Throughout the manuscript, it is not mentioned even once that their CD19-CAR T cells will destroy healthy B cells in patients that do not suffer from B cell leukemia and, hence, have no therapeutic indication of targeting CD19. This bears the risk of life-long B cell aplasia. Also, systemic activation of the infused CAR T cells might cause severe side effects (CRS and CRES) as frequently observed with ALL and LBCL patients.

**Thank you, we have added text and references to the discussion (p 25) to address these points**

2) They authors should discuss why they chose permanent secretion of BPs by genetically modified T cells over infusion as a drug. The latter approach offers several advantages: a) an on/off switch in case side effects (e.g. ‘on-target, off-tumor’ toxicity) occur and b) it would obviate the need to manufacture a new T cell product for each different tumor entity. Opposed to that, their approach lacks both a safety switch and flexibility. In light of this and also to avoid life-long B cell aplasia, use of other safety strategies such as integration of suicide genes or elimination markers should be discussed.

**There are two important points here. First, biologic development and then dosing with CARs is very complicated (see Unum), slow, and costly. Each biologic will have to be IND enabled, and the dose of each relative to each specific antigen will be different – this is a very tough drug development challenge. The secreted BP increases as the CAR T cells proliferate – thus the BP only accumulates where it is needed. Finally the clinical plan is to treat CNS metastatic disease and injected biologics will not cross the BBB.**

**We have added to the discussion some thoughts about on-off tuning of CAR T cells, with references (page 25).**

3) The numbering of the different BP and CAR constructs is utterly confusing and makes it extremely difficult to follow the experiments and results. Why does the ‘empty’ CD19-CAR T cells have an assigned number as well? Sometimes they are referred to by this number, sometimes not. In order to simplify the manuscript the reviewer suggests to re-number the constructs starting from #1, #2, #3 or use a nickname instead of numbers. Also, in the reviewer’s opinion a schematic representation of the BPs and CARs would provide a better (visual) overview than a table.

**Yes, thank you, we agree. We added a graphical Figure (Fig 1) to illustrate the concepts, and have drastically cut down the number of constructs discussed.**

4) The authors constructed a vast amount of different constructs / BP variants which makes the manuscript extremely complex whilst losing focus on the main results. In order to strengthen and shorten the manuscript, the reviewer suggests to select for some constructs (e.g. either ECD-A or ECD-B) and only briefly mention the others that are not relevant for the major message (e.g. #173, #174, #221).

**See above comment please.**

5) All Figures completely lack the number of donors / experiments / mice. Though this information is mentioned in the material and methods section, it should rather be given within the Figure itself (or alternatively the Figure legend). Moreover, the labeling of the figures should be improved in a way that they can be understood without having to read the figure legend, e.g. bar diagrams are constantly missing the legend.

**Thank you, that was my error, we have now added much more information to each experiment.**

6) Restimulation assay:
   - In the results part, it is mentioned that CAR T cells were exposed to target tumor cells for 2 days and “then removed”. However, this is not mentioned in the methods section. Which cells were removed (T cells or target cells)? How was that achieved?

**To be clearer we rewrote this section and also the methods. The target cells are mainly depleted at the time the T cells are washed and rested. Then, more target cells are added. It is meant to be an additive effect over time, as one would encounter within a CAR T cell population.**

- The interpretation of the assay results is extremely difficult and dependent on a lot of variants. Some controls are missing to help interpreting the data. To rule out the influence of using adherent vs suspension cell lines for restimulation, target cells expressing both antigens should have been used (obtained by ectopic expression of CD19 by SKOV3 or Her2 by Raji cells).

**It is not so simple and feasible for our very small lab. We have worked with what we have. However these studies are ongoing and will be the subject of a separate paper on mechanisms in which much more data we will hope to bring to the story. In the meantime we have greatly simplified the data and the interpretation of the data.**

- Representation of the results in Figure 3 is difficult to understand. What exactly was normalized to 100 (should be specified more clearly)? The unit (%) is missing in the labeling. The reviewer strongly disagrees to label the axes with ‘proliferation’ since overall expansion was assessed and not proliferation (which together with apoptosis results in overall increase or decrease of the cell number).

**Please see the above comment and also we have removed the normalization and just present the actual fold-increase.**

- Figure 3C) is difficult to understand. The Figure legend states that CD19 CAR T cells (#254) were restimulated one or three rounds, according to the diagram labeling with either B cells (0>B>B>B>B) or SKOV3 cells (0>S>S>S>S). Yet, the latter does not make sense. What do the authors mean by that? How was restimulation achieved in this case? The same issue occurs for the Her2 CAR T cells (#390) in the right hand part of the Figure (how were these T cells repetitively restimulated with B cells)?

**Please see the above comments - this has been greatly simplified.**

- It would be interesting to assess not only cytotoxic capacity after repetitive restimulation but also other parameters as for example cytokine secretion, exhaustion and memory status of the CAR T cells.

**As noted we are working on the mechanisms. I can say at this point it is not costimulation, cytokines, nor PD-1. We see some hints from the work of Dr R German and Dr M Davis and are pursuing these now, but have no definitive answers yet.**

- Is there a difference in exhaustion between the ‘empty’ CD19 CAR T cells and the ones constantly producing BP?

**No.**

7) *In vivo* experiments:
   - Why did the authors assess *in vivo* half-life of purified BP #42 and not #340 with a stabilized CD19 ECD? What is the point in stabilizing the CD19 ECD when not using it *in vivo*? A comparison of the two BPs would have been interesting. If there is no difference between the two proteins, the authors could have simplified the manuscript by choosing either one or the other construct.

**At the time we only had #42 in quantity as endotoxin clean protein.**

- The mouse experiment testing functionality of the two CD19-CARs secreting the anti-Her2 BP lacks the CD19 CAR T cells (#254) as a control. It would have been interesting to investigate how permanent protein production of CAR T cells does influence their cytotoxic capacity against CD19-expressing target cells *in vivo*.
- The authors should explain why they used #142 (non-stabilized BP) in the mouse experiment shown in Figure 4 D/E but #374 (stabilized BP) in the mouse experiment depicted in Figure 5. Why not always use both? Or select one and not switch?

**These experiments were initiated with prototype forms of the CAR/BP while we decided which programs to advance to the clinic. These in vivo experiments take a long time to run and are very expensive. So there is the early version in the models and the later version in the models.**

- The experimental design of the experiment shown in Figure 5C is difficult to understand. According to the Figure legend CAR T cells that survived the SKOV3 challenge subsequently cleared CD19-positive Nalm-6 leukemia challenge. The diagram shows CAR-254 as control mice that also cleared Nalm-6 tumor challenge. Yet, according to Figure 5B these mice all died.

**Only the naïve control mice challenged with leukemia cells died. The SKOV3 survivors survived the Nalm6 rechallenge**

8) The authors discuss ‘alternative elegant solutions to dual antigen targeting’ (page 26-27) but completely ignore adaptor CAR strategies (e.g. Tag-specific, Fc-binding or bispecific adaptor platforms). They should at least be briefly discussed as well.

**The adaptor CAR strategies are now added and referenced (page 27) see next bullet point.**

9) On page 27, a comparison to T cells secreting BiTEs is drawn. Yet, the important difference is not mentioned that BiTEs, as opposed to CD19 ECD bridging proteins, redirect all T cells and not only the infused genetically modified T cells so that they might possess a broader efficacy

**The bridging protein is a CAR-T cell engager. The bispecific engagers do engage all CD3-positive cells, with varying effects. BiTEs are now discussed and referenced, see page 28.**

10) The authors claim that due to the very short half-life of the BPs, systemic exposure and binding to normal tissues will be limited. This might be the case at the beginning of therapy when the tumor burden is still high. However, once the tumor is cleared, will there be an increased risk of BP binding to healthy antigen-expressing cells?

**In the absence of abundant antigen CARs will gradually diminish to background levels as seen in the leukemia/lymphoma setting; here we expect little accumulation of BP on normal tissue with low antigen expression due to the very short T1/2 of the BP. We have added discussion and a new reference to make this point on page 30.**

Except for the Figure 3, no statistical analysis of the data was performed. Why?

**My oversight - We have added stats throughout.**

Minor issues:

1) Since different Her2-expressing target cell lines (SKOV3, BT474, JeKo-1) were used, it would have been interesting to assess the antigen expression density (molecules per cell). **We have added FACs data throughout.**
2) The composition of the CD19-CAR, particularly which signaling domain is used, should clearly be stated at least in the introduction or the results part and should not be ‘hidden’ in the material and methods section. **We added a line to results page 10.**
3) Table 1 contains an error: construct #311 is ‘Stabilized CD19 ECD-B – anti-Her2’ **Removed**

4) The data on Western Blot analysis of #42 and #340 should be shown as supplementary information instead of stating “data not shown” (page 7) **Removed**
5) It should be clarified whether the effector cell number (E:T ratio) refers to the total number of T cells used or if it was adjusted according to the transduction rate (CAR^+^ T cells). **We used total T cell count throughout, not adjusted for CAR-% percentage. We have added details about the CAR preparations used in the different experiments.**
6) In the reviewer’s opinion, IC_50_ should be changed to EC_50_ since BPs are not antagonistic drugs and don’t possess any inhibitory effect on their own. Instead, the authors assess the BP concentration required for obtaining 50% of a maximum effect, i.e. mediating killing of the tumor cells. **I apologize, but** **we disagree. The IC50 does not vary with E:T ratio, thus the protein concentration controls the inhibition of tumor cell growth/survival. This also allows us to distinguish binding from cytotoxicity concentrations.**
7) The legend in Supplemental Figure S3A and S3C should be shortened, since the well ID (A01/A02/A03 and B12/C12/D12) is not relevant **We have rewritten this.**
8) What is the control in Figure 1B)? The figure legend states that CAR-CD19 T cells as effectors and SKOV3 as target cells were used. However, there should not be seen any cytotoxic effect (diagonal bar).

**We have performed the experiment again, for you, and added new controls, and the new data and increased the ‘n’. Also we have rewritten the legend as there was an error.**

9) Page 14, lines 2/4: “in activated [CAR] T cell culture supernatants” is mentioned twice in the same sentence and should be corrected**. Corrected.**
10) Pages 17/18 – A complete section seems to be iterated by mistake: “… the differences in increase in response to initial Raji cell stimulation versus CAR T cells alone … The cells restimulated with SKOV3 first had not died, and they retained some cytotoxic activity, …” **This has been streamlined and rewritten.**
11) Page 21 – the figure reference is incorrect: “… except that there was the loss of one animal by the end of the study due to tumor burden (Fig. 5c).” Similarly, the text refers to Figure 5d twice, which is not existing. **Thank you, we have corrected this.**
12) Page 25: it is stated that Daudi cells were used as target cells in *in vitro* experiments to show interaction of Her-2 bridging CAR-CD19 T cells with B cell targets. However, the reviewer cannot find the respective data anywhere in the manuscript. **Yes, my mistake, thank you, that has been removed.**
13) The methods section can be shortened massively, e.g. performance of ELISA as a binding detection method is described three times (with only minor variations) as is setting up Luc-based cytotoxicity assays. **Yes, thank you, we did this.**

...................................................................................................................................................................

**Reviewer #3:**

The numbering of CAR constructs and bridging proteins hampers enormously the understanding of the presented data. It is not clear, why the molecules were not numbered in a clear ascending order (#1, #2, #3). For publication, nomenclature must be easier especially for understanding the Figures e.g. by giving short names (e.g. CD19ECDwt-antiHer2, CD19ECDA-antiHer2,…) rather than confusing numbers (even though they are explained in Table 1). In my opinion, the manuscript would benefit a lot from a schematic overview of all CAR constructs and BP. They should be also listed in separate Tables/graphs.

**We agree and have greatly streamlined the manuscript and presentation and have removed non-essential sections such as the aggregation assay data.**

The authors did not mention nor discuss one major limitation of their approach: attack of B cells in tumor patients which will result in long-term B cell aplasia and might also bear the risk for CRS, CRES as seen in B cell lymphoma and leukemia patients.

**B cell aplasia is a well understood and generally readily managed consequence of anti-CD19 CAR T cell therapy, as it is of rituximab therapy etc. There have been no regulatory concerns raised by the agency. We have however added a discussion of B cell aplasia to page 25. CRS and CRES are consequences of any T cell engaging therapeutic (CAR, BiTE) and are not specific to this technology. Also these threats are well understood and better managed now in the clinic (eg. Kymriah label requires 2 doses of tocilizumab on hand). See also the references to preclinical use of dasatinib, page 26.**

Several questions remain concerning assay design and data interpretation:
o page 8: “….the CD19 stabilized bridging proteins #311 and #340 bound SKOV3 cells…” Why do the authors here include #311 and not in the tests before? #311 was also not applied in the following cytotoxicity tests to compare functionality with #42 and #340.

**The nomenclature and use of different constructs has been greatly simplified.**

o Page 9: “…in additional flow cytometry assays (n= 2 to 6 repeats) using Her2-positive SKOV3…” Why was the experiment repeated? Binding results for the bridging proteins to SKOV3 were already presented in Figure S3D. What does “n= 2 to 6 repeats” mean? Please identify how often a certain assay was performed (might be included in Table 2)!
o Figure 1A+B: The authors should include appropriate mock transduced control T cells, to confirm specificity of their system. Furthermore, it is not clear which CAR T cell was used as control in Fig1B (diagonal bars). For cytotoxicity assays with SKOV3, authors should include controls with CD19 CAR T cells + tumor cells without the bridging protein. At which E:T ratios were the assays performed?

**We have added the number of biologic replicates (eg. # of wells) and also the number of times an experiment was repeated, throughout the paper. These sections are rewritten for better clarity. Controls are clearly identified.**

o To characterize functionality of BP-secreting CD19 CAR T cells also cytokine secretion and proliferation/T cell expansion (not only in the context of restimulation) should be analyzed.

**The desired readout was cytotoxicity, not the other parameters.**

o Supplemental Table S1: The authors cannot draw the conclusion that CD19 ECD doesn´t bind to CD21 as the selected cell line Raji naturally expresses CD19 that might already block the CD21 binding side.

**Yes we agree. We added an experiment for you, using OCI-LY3 lymphoma cells. These cells are CD19-negative but CD21 and CD81 positive. The results are added to the Supplemental Table and the flow cytometry data are added in the Supplement Figure.**

o Restimulation assay: How did the authors removed tumor cells from co-cultures to rest CD19 CAR T cells before the next round of restimulation? For data interpretation, it would be of interest to analyze memory phenotype, T cell exhaustion and activation status of CAR T cells during the restimulation process.

**To be clearer we rewrote this section and also the methods. The target cells are mainly depleted at the time the T cells are washed and rested. Then, more target cells are added. It is meant to be an additive effect over time, as one would encounter within a CAR T cell population. We are working on the mechanisms. I can say at this point it is not costimulation, cytokines, nor PD-1. We see some hints from the work of Dr R German and Dr M Davis and are pursuing these now, but have no definitive answers yet. This will be addressed in an upcoming paper.**

o Figure 3C shows that αHer2 CAR T cells do not lose cytotoxic capacity against SKOV3, after restimulation with B cell lines, but after restimulation with SKOV3. This indicates that the cell lines used for restimulation may provide “general” stimulatory or inhibitory signals to all CAR T cells (independent of their specificity). To analyze this in more detail, not restimulated CAR T cells should be included into the cytotoxicity assays. In addition, all restimulation assays should be performed with other CD19+ and Her2+ cells. It would be even more relevant to show that HLA-matched human B cells from peripheral blood (and not an artificial cell lines) are able to restimulate CD19 CAR T cells as this reflects the situation in the patients.

**CAR T cells are prepared and stored until use. Donor B cells will not freeze well at all, and die overnight in culture. We are working with additional cell lines as we investigate mechanism. We would love to do the B cell experiments as you describe but it is logistically very difficult for us.**

o Cytotoxicity assay with the dual bridging protein anti-Her2-CD19ECD-anti-EGFR was performed in comparison to #311. Why did the authors not selected #42 as control. This would be more reasonable as #460 was constructed on the basis of #42. Selection of different CD19ECD variants might influence overall performance redirected CD19 CAR T cells. No data are available showing that #311 and #42 mediate comparable tumor lysis.

**We agree and have greatly streamlined the manuscript and presentation. Different reagents were available over the course of the study. The difference in CD19 ECD is mainly in the amount of BP expression achieved.**

o Why was in vivo serum half-life only analyzed for #42, but not for #340? Why do authors switch between #142 and #374 during their experiments?
o In vivo functionality of BP-expressing CARs (#142, #374) should be compared to a group that received CD19 CAR T cells (#254) and BP (#42, #340) as infusion.

**We only had #42 in a large endotoxin-free batch at that time. We have performed the in vivo experiments that we could including the CAR-CD19 (254) controls – the addition of purified #42 and #340 is not useful as the half-life is very short. We are making a half-life enhanced form of 340 now (adding an anti-albumin VHH to keep it monomeric).**

Data presentation:
• Statistical analysis is missing in most of the Figures. **Added throughout**
• Please indicate number of performed experiments. **Added throughout**
• Abbreviations used in the Figures should be explained in the figure caption e.g. NA, UTD **Added throughout**
• Table 1: #311 description is incorrect **removed**
• Figure 1C/D: It is stated that a “fixed E:T ratio” was used, please indicate exactly at which E:T ratio the assay was performed 10:1, 3:1, or 1:1? **Added throughout**

• Page 7: “When the two bridging protein #42 and #340, were tested…in an ELISA (FMC63 capture, Her2-Fc detection)…” --> In Table 2, combination of FMC63 capture + Her2-Fc detection is indicated as “ND” by the authors. What was actually done? Are these data presented in Supplemental Figure S2? If so, authors should also refer to the Figure in the main text. **Table has been updated with new data, so in some cases n is now larger and in other cases we have added data.**
• Figure 5 consists of three panels (a-c). However, in the text the authors refer to Figure 5d that does not exist. **Redone**
• Figure S3B: Authors state that binding affinity of #42 and #340 is significantly different. **Only for binding to CD19, not on the other end (anti-Her2). The differences are now stated more clearly in the text, on page 6 and 7.**
Please indicate test and significance level in figures **statistics added throughout**
What does MW(Kd) mean? Do the authors here refer to “Kilodalton = kDa”?
• Figure S3A/C: What is the purpose of adding A03, A02, A01 or D12, C12, B12 in the figure
legend? **Removed.**

Discussion:
• Why is the secretion of BP via CD19 CAR T cells beneficial compared to BP infusions? With regard to “on-target, off-tumor” effects, the system …may necessitate tuning of CAR affinities…”, as stated by the authors. Multi-specific tumor-targeting with CD19 CAR T cells secreting BP will also require production of several T cell products from the same patients.

**No, not true, and this is important to understand – we make multi-antigen bridging proteins, eg. anti-Her2-CD19-anti-EGFR can be cloned into the lentiviral system and it is a single CAR T cell secreting the multi-antigen BP**

• The proposed approach should be compared to other adaptor CAR systems described in the literature.

**Added in discussion, with references, page** **27**

**Reviewer #4:**

the authors do not discuss the pro and cons of their approach in light of published BiTE and switch CAR-T approaches.

**Added in discussion, with references, pages 27-28**

Result section
Autoactivation of CD19 CAR-T by the soluble binding proteins is a critical issue for the system. Data (e.g. Absence of cytokine secretion) should be provided to demonstrate the safety of the system as claimed in the introduction and discussion section.

**This is specifically addressed in the order of addition assay.**

Table 2. Indicate on how many repeated experiments and technical repeats the affinity determination is based on. **This information is added throughout**

Stability at 37°C is only relevant if demonstrated in presence of human serum – was human serum present? Please comment. **Yes, but we have now removed this data** **from the paper.**

Determined half-life of the bridging proteins is ~ twice as long as the half-life of clinical bispecific antibodies (e.g. Blinatumomab). Maybe the ELISA used is only sensitive for dimers? Was the impact of mouse serum on the ELISA evaluated? The authors should comment on this.

**Half-life calculation is now presented. T1/2 is ~60 minutes. There are no dimers, the protein is a monomer.**

The used coding system for the CARs and bridging proteins makes it difficult to follow the manuscript. The reviewer suggests to replace it by using short names indicating antigen combination, e.g. #42 = CD19-HER2. In all figures the number of tested donors and mice are missing. The information is provided in the M&M section, but should be included in the figure legends.

**We have greatly streamlined the paper to simplify the nomenclature, and have followed your suggestion as to naming both in the text and in the figures. We have added the number of biologic replicates (eg. # of wells) and also the number of times an experiment was repeated, throughout the paper.**

Please comment on the high background lysis of non-transduced cells in Fig. 1 and 2. In Fig. 2 the UTD control seems to be missing.

**UTD are ‘noisy’ depending on the donor. This is due to homeostatic proliferation and possibly allo reactivity. The figures are redone.**

Figure 4 is miss-labelled as Figure 5 **Thank you, this is fixed**
Statistical analysis of the in vivo data is missing. **Added, this was my oversight.**
The re-challenge in vivo experiment is based on a very small number of mice, expanding the groups would strengthen the data set.

**We agree but are now “flipping” the order: giving the Nalm6 first and then the SKOV3. We also tried some experiments adding donor matched B cells but these seem not to survive in the mice.**

Discussion section
Targeting less differentiated antigens beyond CD19, CD22, or BCMA bears the risk of on target/of tumor toxicities. The described approach would rely on continuous secretion of the bridging protein. How does the authors envisioned to avoid on target/off tumor toxicities? Claimed “limited systemic exposure” is very hypothetical.

**The claimed “limited systemic exposure” is supported by the references cited, specifically the Maus lab paper. We have expanded the clinical application section in the discussion to specifically address this point - page 29.**

In particular the reviewer would expect sever toxicities for a clinical approach of the HER2-specific adaptor due to a first pass effect in the lung as seen with CAR-T harboring the same binder (Morgan et al. 2010).

**See above comment. Also, this single clinical experiment from 2010 used very high chemo doses and very high infused cell number – the method has little resemblance to current protocols used in cell therapy.**

The approach hazard the consequences of a continued B cell aplasia, which is clinical manageable, however bears great health risks for patients, comes along with high costs and has a severe adverse impact on the quality of life of patients. The authors should comment how they envision implementing such an approach into the clinical practice.

**We added a section on B cell aplasia to the discussion, page 26, with references.**

In the light of these arguments the approach falls back beyond switch CAR-T approaches published by several groups, in which the CAR binding moiety does not recognize a surface protein. The authors should at least cite the most relevant publications from these field and compare their approach to these earlier established systems of which some are already clinical evaluated.

**This is in the discussion now pages 27ff, with references added.**

Minor comments:
p. 8 Donor human T cells were transduced with lentiviral particles that express the scFv derived from the anti-CD19 antibody FMC63 (CAR-CD19, encoded by construct #254, Supplemental Fig. 3a). -> Please clarify that the lentiviral vector encoded transgene is a CAR harboring the scFv derived from the anti-CD19 antibody FMC63
Some typing errors throughout, e.g.
p. 13: Bindng of CD19-anti-Her2 bridging proteins
p. 25: In the in vivo model, serial presentation of antigens (Her2, CD19) showed that the Her2-bridging CAR-CD19 T cells could ehgage both cell types.

**Fixed, thank you.**
