# Supplementary material for: Anti-CD19 CAR T cells potently redirected to kill solid tumor cells
Source: PLoS One. 2021 Mar 18;16(3):e0247701. doi: 10.1371/journal.pone.0247701 (PMC7971483; doi:10.1371/journal.pone.0247701)
Supplement: Attachment — Submitted filename: Reviewer comment for CD19-CAR paper.docx [file pone.0247701.s012.docx]

In their submitted manuscript, Ambrose et al. suggest the use of anti-CD19 CAR T cells permanently expressing a bridging protein, e.g. targeting Her2, for therapy of solid tumors. This is an interesting approach since CAR T cell therapy for solid tumors still fails to live up to expectations due to several reasons, one being poor T cell persistence. However, there are several important issues that need to be addressed and the manuscript needs to be extensively revised before it can be considered for publication.

Major points:

1) The authors should discuss major drawbacks of their approach. Throughout the manuscript, it is not mentioned even once that their CD19-CAR T cells will destroy healthy B cells in patients that do not suffer from B cell leukemia and, hence, have no therapeutic indication of targeting CD19. This bears the risk of life-long B cell aplasia. Also, systemic activation of the infused CAR T cells might cause severe side effects (CRS and CRES) as frequently observed with ALL and LBCL patients.
2) They authors should discuss why they chose permanent secretion of BPs by genetically modified T cells over infusion as a drug. The latter approach offers several advantages: a) an on/off switch in case side effects (e.g. ‘on-target, off-tumor’ toxicity) occur and b) it would obviate the need to manufacture a new T cell product for each different tumor entity. Opposed to that, their approach lacks both a safety switch and flexibility. In light of this and also to avoid life-long B cell aplasia, use of other safety strategies such as integration of suicide genes or elimination markers should be discussed.
3) The numbering of the different BP and CAR constructs is utterly confusing and makes it extremely difficult to follow the experiments and results. Why does the ‘empty’ CD19-CAR T cells have an assigned number as well? Sometimes they are referred to by this number, sometimes not. In order to simplify the manuscript the reviewer suggests to re-number the constructs starting from #1, #2, #3 or use a nickname instead of numbers. Also, in the reviewer’s opinion a schematic representation of the BPs and CARs would provide a better (visual) overview than a table.
4) The authors constructed a vast amount of different constructs / BP variants which makes the manuscript extremely complex whilst losing focus on the main results. In order to strengthen and shorten the manuscript, the reviewer suggests to select for some constructs (e.g. either ECD-A or ECD-B) and only briefly mention the others that are not relevant for the major message (e.g. #173, #174, #221).
5) All Figures completely lack the number of donors / experiments / mice. Though this information is mentioned in the material and methods section, it should rather be given within the Figure itself (or alternatively the Figure legend). Moreover, the labeling of the figures should be improved in a way that they can be understood without having to read the figure legend, e.g. bar diagrams are constantly missing the legend.
6) Restimulation assay:
   - In the results part, it is mentioned that CAR T cells were exposed to target tumor cells for 2 days and “then removed”. However, this is not mentioned in the methods section. Which cells were removed (T cells or target cells)? How was that achieved?
   - The interpretation of the assay results is extremely difficult and dependent on a lot of variants. Some controls are missing to help interpreting the data. To rule out the influence of using adherent vs suspension cell lines for restimulation, target cells expressing both antigens should have been used (obtained by ectopic expression of CD19 by SKOV3 or Her2 by Raji cells).
   - Representation of the results in Figure 3 is difficult to understand. What exactly was normalized to 100 (should be specified more clearly)? The unit (%) is missing in the labeling. The reviewer strongly disagrees to label the axes with ‘proliferation’ since overall expansion was assessed and not proliferation (which together with apoptosis results in overall increase or decrease of the cell number).
   - Figure 3C) is difficult to understand. The Figure legend states that CD19 CAR T cells (#254) were restimulated one or three rounds, according to the diagram labeling with either B cells (0>B>B>B>B) or SKOV3 cells (0>S>S>S>S). Yet, the latter does not make sense. What do the authors mean by that? How was restimulation achieved in this case? The same issue occurs for the Her2 CAR T cells (#390) in the right hand part of the Figure (how were these T cells repetitively restimulated with B cells)?
   - It would be interesting to assess not only cytotoxic capacity after repetitive restimulation but also other parameters as for example cytokine secretion, exhaustion and memory status of the CAR T cells. Is there a difference in exhaustion between the ‘empty’ CD19 CAR T cells and the ones constantly producing BP?
7) *In vivo* experiments:
   - Why did the authors assess *in vivo* half-life of purified BP #42 and not #340 with a stabilized CD19 ECD? What is the point in stabilizing the CD19 ECD when not using it *in vivo*? A comparison of the two BPs would have been interesting. If there is no difference between the two proteins, the authors could have simplified the manuscript by choosing either one or the other construct.
   - The mouse experiment testing functionality of the two CD19-CARs secreting the anti-Her2 BP lacks the CD19 CAR T cells (#254) as a control. It would have been interesting to investigate how permanent protein production of CAR T cells does influence their cytotoxic capacity against CD19-expressing target cells *in vivo*.
   - The authors should explain why they used #142 (non-stabilized BP) in the mouse experiment shown in Figure 4 D/E but #374 (stabilized BP) in the mouse experiment depicted in Figure 5. Why not always use both? Or select one and not switch?
   - The experimental design of the experiment shown in Figure 5C is difficult to understand. According to the Figure legend CAR T cells that survived the SKOV3 challenge subsequently cleared CD19-positive Nalm-6 leukemia challenge. The diagram shows CAR-254 as control mice that also cleared Nalm-6 tumor challenge. Yet, according to Figure 5B these mice all died.
8) The authors discuss ‘alternative elegant solutions to dual antigen targeting’ (page 26-27) but completely ignore adaptor CAR strategies (e.g. Tag-specific, Fc-binding or bispecific adaptor platforms). They should at least be briefly discussed as well.
9) On page 27, a comparison to T cells secreting BiTEs is drawn. Yet, the important difference is not mentioned that BiTEs, as opposed to CD19 ECD bridging proteins, redirect all T cells and not only the infused genetically modified T cells so that they might possess a broader efficacy.
10) The authors claim that due to the very short half-life of the BPs, systemic exposure and binding to normal tissues will be limited. This might be the case at the beginning of therapy when the tumor burden is still high. However, once the tumor is cleared, will there be an increased risk of BP binding to healthy antigen-expressing cells?
11) Except for the Figure 3, no statistical analysis of the data was performed. Why?

Minor issues:

1) Since different Her2-expressing target cell lines (SKOV3, BT474, JeKo-1) were used, it would have been interesting to assess the antigen expression density (molecules per cell).
2) The composition of the CD19-CAR, particularly which signaling domain is used, should clearly be stated at least in the introduction or the results part and should not be ‘hidden’ in the material and methods section.
3) Table 1 contains an error: construct #311 is ‘Stabilized CD19 ECD-B – anti-Her2’
4) The data on Western Blot analysis of #42 and #340 should be shown as supplementary information instead of stating “data not shown” (page 7).
5) It should be clarified whether the effector cell number (E:T ratio) refers to the total number of T cells used or if it was adjusted according to the transduction rate (CAR^+^ T cells).
6) In the reviewer’s opinion, IC_50_ should be changed to EC_50_ since BPs are not antagonistic drugs and don’t possess any inhibitory effect on their own. Instead, the authors assess the BP concentration required for obtaining 50% of a maximum effect, i.e. mediating killing of the tumor cells.
12) The legend in Supplemental Figure S3A and S3C should be shortened, since the well ID (A01/A02/A03 and B12/C12/D12) is not relevant.
13) What is the control in Figure 1B)? The figure legend states that CAR-CD19 T cells as effectors and SKOV3 as target cells were used. However, there should not be seen any cytotoxic effect (diagonal bar).
14) Page 14, lines 2/4: “in activated [CAR] T cell culture supernatants” is mentioned twice in the same sentence and should be corrected.
15) Pages 17/18 – A complete section seems to be iterated by mistake: “… the differences in increase in response to initial Raji cell stimulation versus CAR T cells alone … The cells restimulated with SKOV3 first had not died, and they retained some cytotoxic activity, …”
16) Page 21 – the figure reference is incorrect: “… except that there was the loss of one animal by the end of the study due to tumor burden (Fig. 5c).” Similarly, the text refers to Figure 5d twice, which is not existing.
17) Page 25: it is stated that Daudi cells were used as target cells in *in vitro* experiments to show interaction of Her-2 bridging CAR-CD19 T cells with B cell targets. However, the reviewer cannot find the respective data anywhere in the manuscript.
18) The methods section can be shortened massively, e.g. performance of ELISA as a binding detection method is described three times (with only minor variations) as is setting up Luc-based cytotoxicity assays.
